# Optogenetic monitoring identifies phosphatidylthreonine-regulated calcium homeostasis in *Toxoplasma gondii*

**DOI:** 10.15698/mic2016.05.500

**Published:** 2016-05-02

**Authors:** Arunakar Kuchipudi, Ruben D. Arroyo-Olarte, Friederike Hoffmann, Volker Brinkmann, Nishith Gupta

**Affiliations:** 1 Humboldt University, Berlin, Germany.; 2 Max-Planck Institute for Infection Biology, Berlin, Germany.

**Keywords:** Toxoplasma gondii, phosphatidylthreonine, optogenetics, gene-encoded calcium indicator, lytic cycle, intracellular parasite, calcium homeostasis

## Abstract

*Toxoplasma gondii* is an obligate intracellular parasite, which inflicts acute as well as chronic infections in a wide range of warm-blooded vertebrates. Our recent work has demonstrated the natural occurrence and autonomous synthesis of an exclusive lipid phosphatidylthreonine in *T. gondii*. Targeted gene disruption of phosphatidylthreonine synthase impairs the parasite virulence due to unforeseen attenuation of the consecutive events of motility, egress and invasion. However, the underlying basis of such an intriguing phenotype in the parasite mutant remains unknown. Using an optogenetic sensor (gene-encoded calcium indicator, GCaMP6s), we show that loss of phosphatidylthreonine depletes calcium stores in intracellular tachyzoites, which leads to dysregulation of calcium release into the cytosol during the egress phase of the mutant. Consistently, the parasite motility and egress phenotypes in the mutant can be entirely restored by ionophore-induced mobilization of calcium. Collectively, our results suggest a novel regulatory function of phosphatidylthreonine in calcium signaling of a prevalent parasitic protist. Moreover, our application of an optogenetic sensor to monitor subcellular calcium in a model intracellular pathogen exemplifies its wider utility to other entwined systems.

## INTRODUCTION

*Toxoplasma gondii* is considered as one of the most successful parasites on Earth, infecting humans as well as a wide range of animals 1. The parasite causes debilitating opportunistic infections in individuals with inadequate and compromised immunity, such as neonates, HIV-AIDS and organ transplantation patients. Acute disease is due to asexual reproduction of the fast-dividing tachyzoite stage of *T. gondii*, which undergoes recurring lytic cycles in the target host cells. The lytic cycle of tachyzoites comprises the sequential events of invasion, replication and egress 1. Our recent work has identified an atypical phospholipid in tachyzoites designated as phosphatidylthreonine (PtdThr) that is crucial for an efficient lytic cycle and virulence 2. PtdThr is produced by a novel enzyme PtdThr synthase (PTS) located in the endoplasmic reticulum (ER) of the parasite. Surprisingly, the loss of PtdThr in a mutant of lipid synthesis (Δ*tgpts* strain) does not affect membrane biogenesis and intracellular replication but compromises the gliding motility, which in turn blights the downstream events of parasite egress and invasion.

The induction of gliding motility in *T. gondii* tachyzoites requires a calcium-mediated signaling cascade leading to activation of calcium-dependent kinases and exocytosis of micronemes once the proliferation phase is completed 3,4,5,6,7,8. The level of calcium in the cytosol is elevated prior to initiation of the parasite egress from dilapidated host cells 9,10. Given the phenotype of the Δ*tgpts* strain, we postulated a role of PtdThr in governing subcellular calcium homeostasis during the lytic cycle of *T. gondii*. Calcium-regulated processes in *T. gondii* occur in an entwined system, comprising the parasite and host cell. While the use of calcium-sensitive fluorophores has proven extremely valuable to examine calcium homeostasis in extracellular stage of apicomplexan parasites 11, similar studies during intracellular development are rather limited 12,13. Specific real-time monitoring of subcellular calcium has become more feasible using optogenetic sensors, which have aided detection of calcium in specific cell types, populations or even organelles 14,15,16. A rich repertoire of gene-encoded calcium indicators (GECI) have been engineered by fusing different types of calcium-binding and regulatory domains with fluorescent proteins to meet the desired specificity, sensitivity, signal-to-noise ratio and temporal scale 14,15,16. For instance, GCaMP6 family proteins consist of a myosin light chain kinase domain (M13) conjugated to a circularly permuted enhanced GFP (CpEGFP) and calmodulin (CaM) 14. A rise in calcium induces conformational change in the CaM domain, which in turn activates the M13 kinase domain to block a hole in the CpEGFP barrel exposing the otherwise-quenched chromophore 17. This work deployed a member of GCaMP6 family, GCaMP6s, to discern the functional importance of PtdThr by examining the calcium dynamics in intracellular tachyzoites.

## RESULTS AND DISCUSSION

### GCaMP6s localizes in the parasite cytosol.

**Figure 1:**
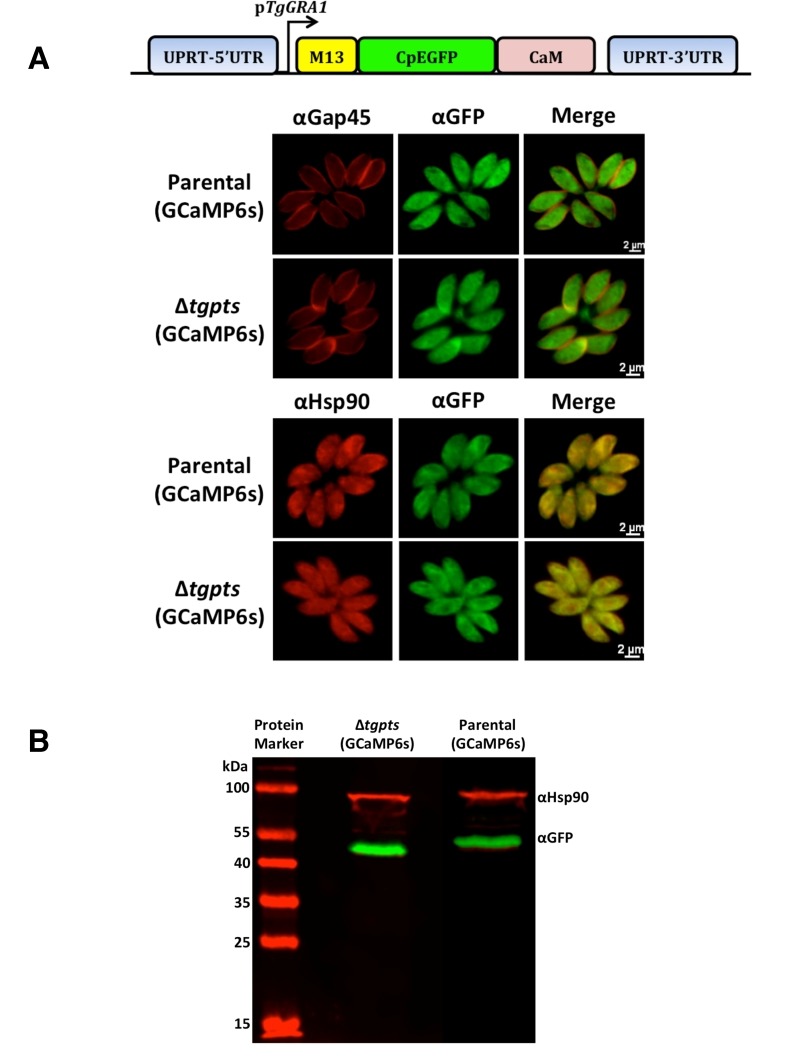
FIGURE 1: GCaMP6s is expressed in the cytosol of intracellular tachyzoites. **(A)** Images illustrating co-staining of EGFP (GCaMP6s) with the markers for cytosol (*Tg*Hsp90) or inner membrane complex (*Tg*Gap45). Stable transgenic parasites of the parental (RHΔ*ku80-hxgprt*-) and PTS mutant (Δ*tgpts*) strains, expressing GCaMP6s under the control of the *Tg*GRA1 promoter, were generated by negative selection with FUDR (targeting the UPRT locus). Intracellular tachyzoites (MOI, 1; 24 h infection) were imaged following the immunofluorescence assay using the indicated antibodies. No cross-fluorescence (bleeding effect) was observed across the two color channels. **(B)** A representative immunoblot confirming the expression of intact M13-CpEGFP-CaM fusion protein (a.k.a. GCaMP6s or GECI) in the parental and PTS knockout strains. *Tg*Hsp90 was used as the loading control.

To determine the calcium oscillation in intracellular tachyzoites, we deployed a gene-encoded calcium indicator GCaMP6s, which was originally designed to image the neuronal activity 14. We generated the parental and PTS-knockout strains stably expressing the GCaMP6s fusion protein, which consists of a M13 kinase peptide, a CpEGFP barrel and a calcium-sensitive calmodulin (Fig. 1A). Transgenic parasites were subjected to immunofluorescence assays to examine the subcellular location and relative expression of GCaMP6s in the two strains (Fig. 1A). GCaMP6s signal was confined within the parasite periphery, as shown by co-staining of EGFP domain with the *Tg*Gap45 protein located in the inner membrane complex 18. Co-localization of the EGFP domain with a cytosolic marker *Tg*Hsp90 19 revealed an apparently uniform distribution of GCaMP6s in the parasite cytosol. Immunoblot analysis demonstrated the expression of a protein band with an apparent molecular weight of about 55 kDa (Fig. 1B), which confirmed the physical integrity of GCaMP6s fusion protein in both strains. Taken together, these data provided the initial basis to monitor cytosolic calcium in tachyzoites.

### Expression of GCaMP6s is not detrimental to the parasite growth and replication.

**Figure 2:**
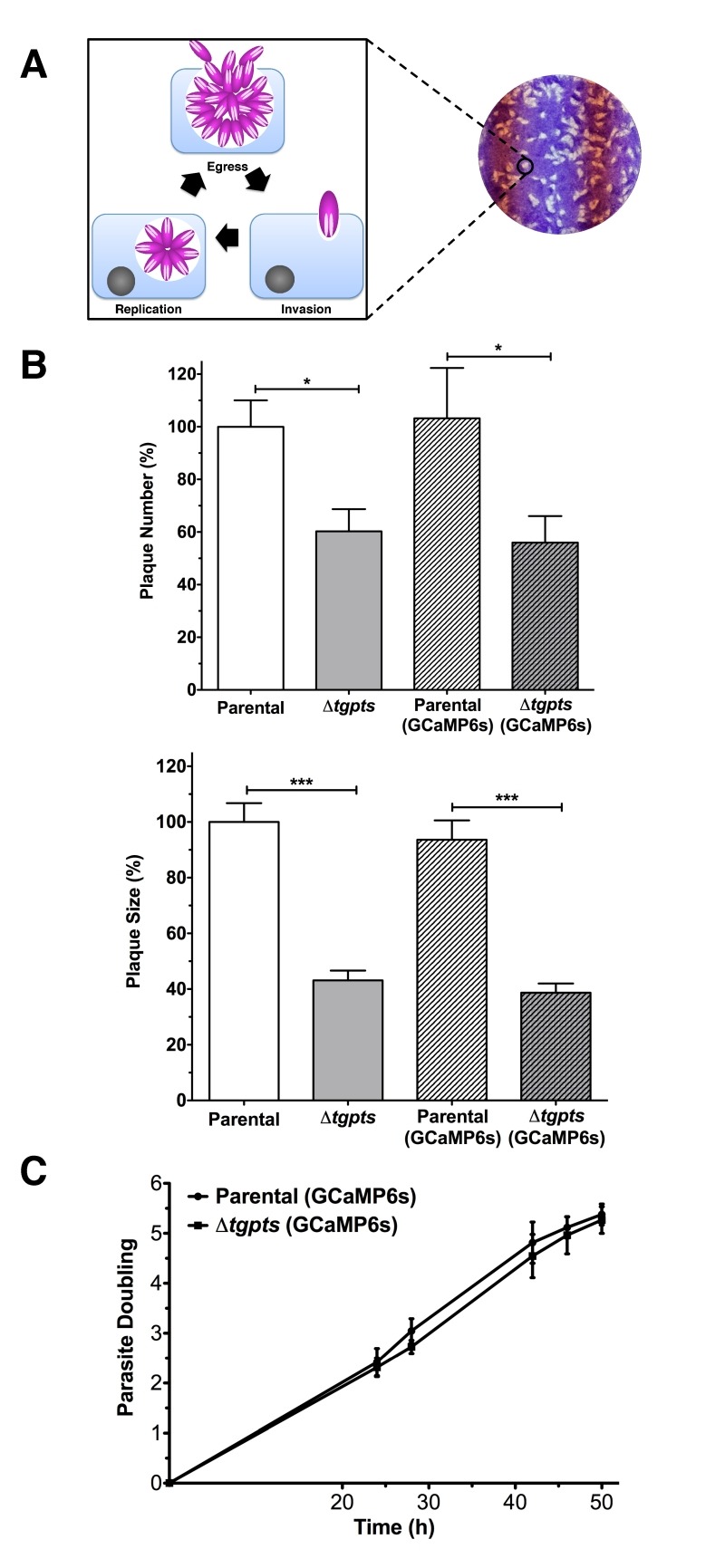
FIGURE 2: *In vitro* growth and replication rates of GCaMP6s-expressing parasite strains are comparable to the respective parental strains. **(A)** Image illustrating the lytic cycle of tachyzoites leading to plaque formation in host-cell monolayers. **(B)** Growth fitness of the specified strains, as measured by plaque assays. In total, 100-200 plaques of each strain were evaluated for their sizes and numbers using ImageJ suite. **(C)** Replication rates of the GCaMP6s-expressing strains. Intracellular tachyzoites proliferating in their vacuoles were immunostained with α*Tg*Gap45 antibody at the indicated time points. The average number of tachyzoites/vacuole was used to calculate parasite doublings (Example: 1 doubling = 2 parasites/vacuole, and 5 doublings = 32 parasites/vacuole). Graphs depict the mean ± SEM from 3 assays (student’s t-test, *p<0.05, ***p<0.001).

Next, we determined the impact of GCaMP6s expression on the parasite growth by plaque assays, which signify consecutive lytic cycle of tachyzoites in host cells (Fig. 2A). As reported previously 2, the Δ*tgpts* mutant displayed a considerably reduced plaque numbers and area compared to the parental strain (Fig. 2B). More importantly, expres-sion of GECI did not exert an apparent negative impact on the growth of either of the GCaMP6s-transgenic strains when compared to their corresponding reference (non-GECI) strains. To reinforce these data, we measured intra-cellular replication of the indicated strains by scoring the number of tachyzoites per vacuole throughout the lytic cycle. The replication rates of respective GCaMP6s-transgenic strains were comparable during the entire pro-liferation phase (Fig. 2C). Collectively, these results excluded a detrimental effect of GECI for tachyzoite growth and enabled the study of calcium dynamics during the intracellular development of the two strains.

### GCaMP6s fluorescence is increased during parasite egress.

**Figure 3:**
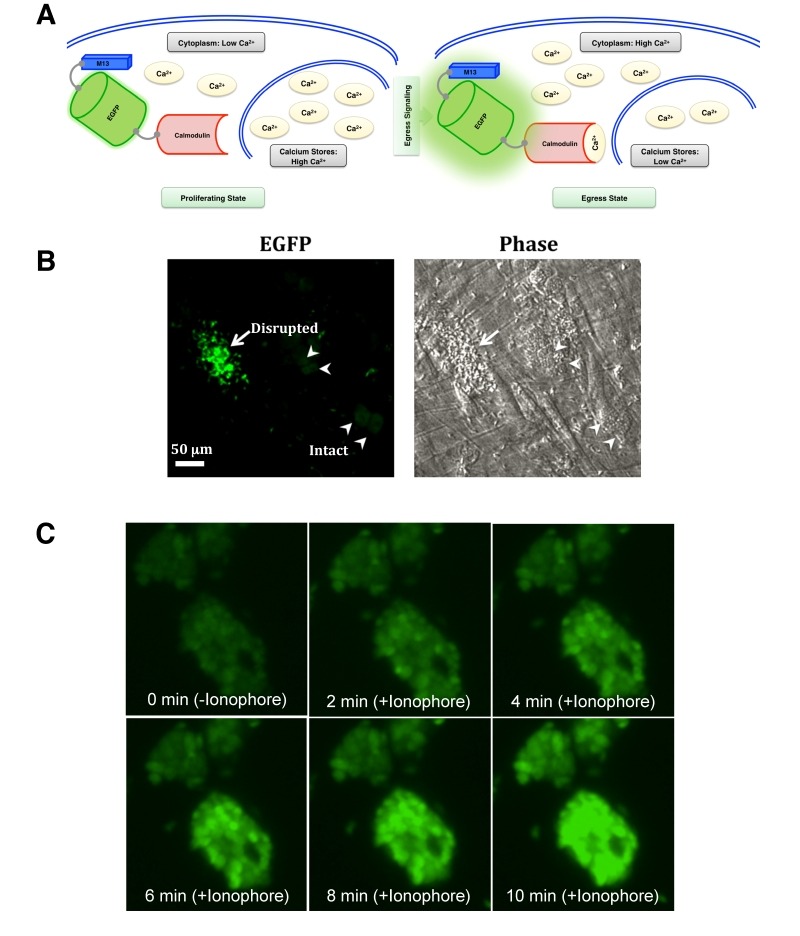
FIGURE 3: GCaMP6s activity in intracellular parasites is induced by natural egress and ionophore. **(A)** Schematics depicting a rise in cytosolic calcium prior to and/or during egress phase, and detection of calcium by GCaMP6s expressed in the parasite cytosol. The effect of external calcium sources is not illustrated here. **(B)** GCaMP6s fluorescence in response to the natural egress. Parasitized cells infected with GECI-transgenic parental strain (MOI, 3; 44 h infection) was subjected to EGFP imaging. Image shows intensified EGFP fluorescence during egress (arrow) and background signal in the intact/immature vacuoles (arrowheads). **(C)** Monitoring of GCaMP6s signal in the parasite cultures treated with ionophore (5 µM A23187). Host cells infected with the parental strain were imaged for 10 min using time-lapse microscopy. Ionophore was added after 1 min of imaging. Still images (snapshots), each with about 2 min intervals, are shown.

Having established the relative expression and growth parameters of the GCaMP6s-transgenic strains, we next examined the functionality of GCaMP6s in tachyzoites. As illustrated in Fig. 3A, tachyzoites egressing at the end of lytic cycle exhibit induced cytosolic calcium levels, which exert conformational changes in EGFP, leading to increased fluorescence. To examine the modulation of GCaMP6s activity during natural egress, we monitored EGFP signal in tachyzoites nearing the end of lytic cycle. Certainly, as anticipated, parasites exiting from dilapidated human host cells displayed an intense EGFP signal, whereas fluorescence in the immature vacuoles was notably lower (Fig. 3B). We endorsed these results by time-lapse microscopy of the parasite vacuoles exposed to an ionophore A23187 (Fig. 3C), which is known to induce calcium influx and consequently the premature egress of intracellular tachyzoites 3,20,21,22. As expected, tachyzoites within vacuoles displayed basal level of fluorescence prior to ionophore treatment (0 min). EGFP signal increased rapidly within a minute of drug exposure and saturated gradually over a period of 10 min. Together with aforesaid assays, these data firmly established the utility of GECI to measure and compare the cytosolic calcium levels in intracellular parasites of the parental and mutant strains. The method also offered other desired benefits, including spatiotemporal detection, high signal/noise ratio, genetic inheritability to the progeny and uniformity of calcium-dependent GCaMP6s signal across the parasite population in parasitized cultures.

### The Δ*tgpts* mutant shows a compromised calcium flux during the parasite egress.

**Figure 4:**
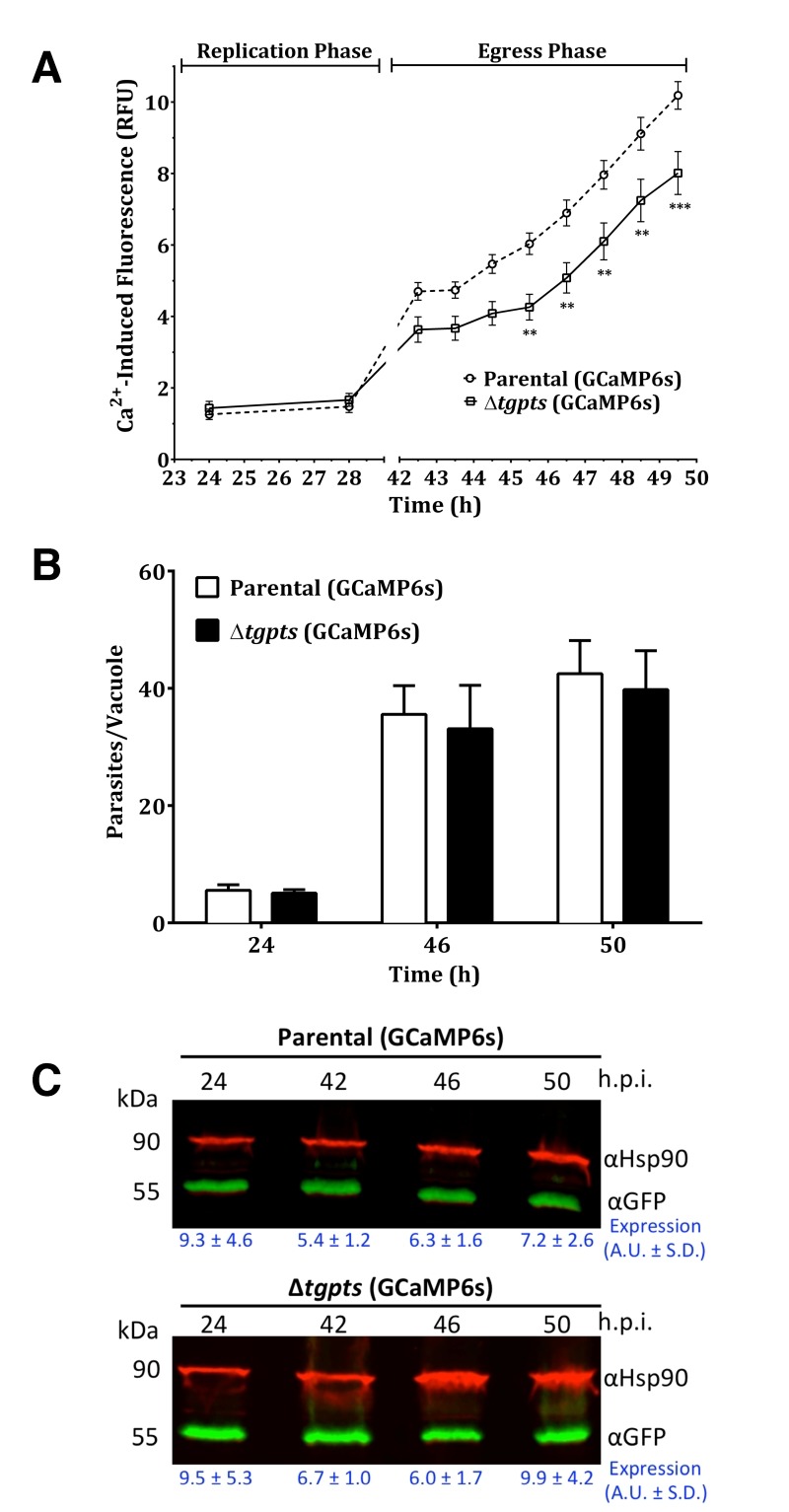
FIGURE 4: The PTS-knockout mutant exhibits a dysregulation of cytosolic calcium during its natural egression but not during the proliferation phase. **(A)** Quantitation of EGFP-derived fluorescence during replication and egress phases of GECI strains. Human fibroblast cultures were infected with GCaMP6s-transgenic parental or Δ*tgpts* strains. Calcium-induced EGFP intensity was measured in living cultures using a microplate reader. Graphs show the mean ± SEM of 9 assays, each in triplicates (student’s t-test, **p< 0.01, ***p<0.001). **(B)** Replication rates of the parental and mutant strains expressing GECI. Intracellular parasites replicating in their vacuoles were quantified at specified time points (representing replication, early and late egress) after immunostaining with anti-*Tg*Gap45 antibody (mean ± SEM, n=3 assays). Consistent with parasite counts, EGFP signal during the replication phase was indistinguishable in the two GCaMP6s-transgenic strains. **(C)** A representative western blot depicting the expression of M13-CpEGFP-CaM in the two parasite strains. *Tg*Hsp90 was used for normalizing the expression of GCaMP6s in each lane.

To investigate calcium homeostasis in the PTS mutant, we employed an automated microplate-based monitoring of EGFP fluorescence. The assay was set up to quantify the real-time levels of cytosolic signal in intracellular tachyzoites of the parental and Δ*tgpts* strains expressing GCaMP6s (Fig. 4A). We observed a comparable level of cytosolic fluorescence during the entire replication phase of the parental and mutant strains, which confirmed a very similar functional activity of GCaMP6s in both strains. The calcium-dependent EGFP fluorescence was progressively elevated as they approached the end of their lytic cycles (42-50 h infection, Fig. 4A). Quite noticeably, the Δ*tgpts* mutant showed a significantly diminished EGFP signal compared to the parental strain during the egress period, indicating a defective release of calcium into the parasite cytosol. To strengthen our results, we numerated intracellular parasites in the two strains at three relevant time points corresponding to replication, early and late egress phases (Fig. 4B). The numbers of intracellular parasites in both GCaMP6s strains were nearly identical at all three time points. Consistently, equivalent intensity of EGFP fluorescence during replication phase reflected a similar number of vacuoles in both strains. Last but not least, immunoblots indicated a comparable expression of GCaMP6s in both strains at specified time points (Fig. 4C). In conclusion, these data suggest a defective mobilization of calcium into the cytosol of the PTS mutant. 

### The Δ*tgpts* mutant exhibits a poor storage of calcium in the parasite.

**Figure 5:**
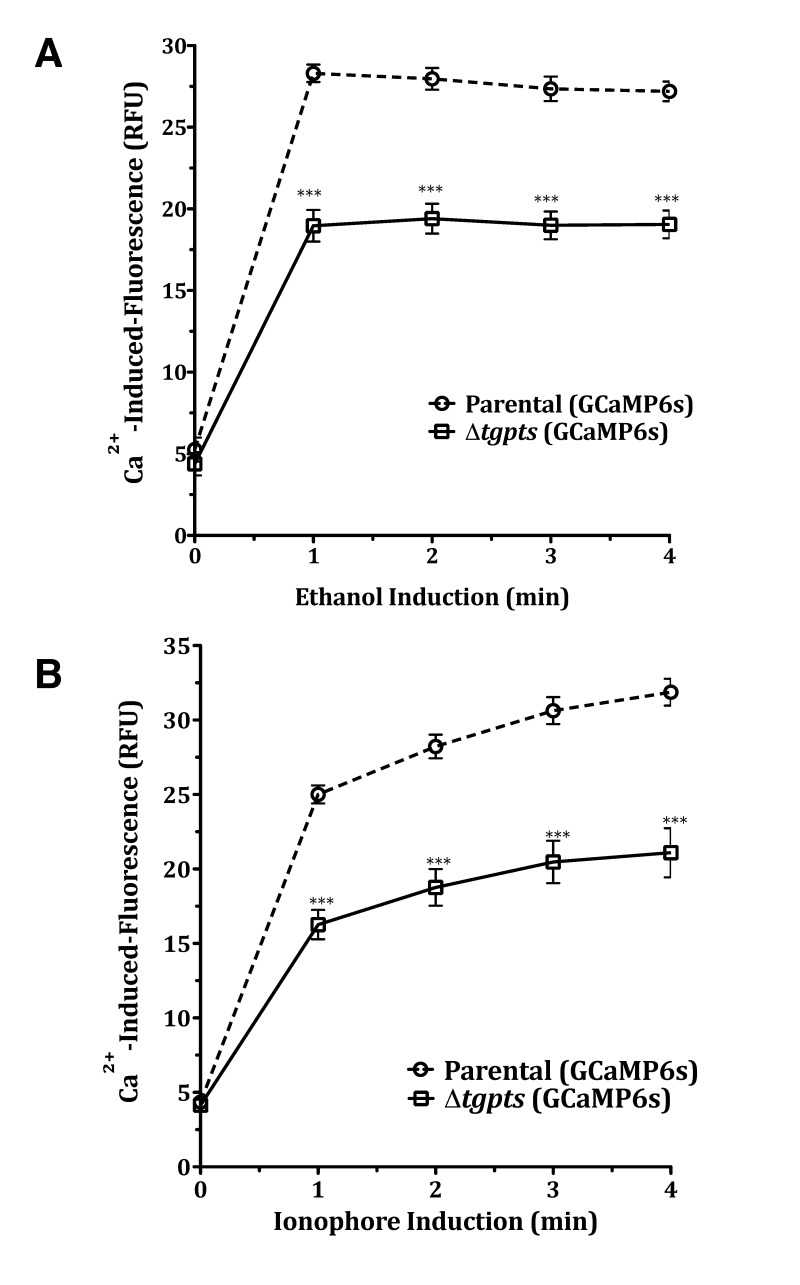
FIGURE 5: The Δ*tgpts* strain is deficient in the storage of subcellular calcium. Calcium-induced fluorescence in the two GECI-strains following exposure to 8 % ethanol **(A)** or to 5 µM A23187 **(B)** (42 hrs post-infection). Human fibroblasts were infected with transgenic parental or Δ*tgpts* strains. As shown in Fig. 4B, the number of intracellular tachyzoites was equivalent in the two strains. Note that EGFP fluorescence signals just prior to drug exposure (0 min) are nearly equal, indicating a comparable number of vacuoles (and thus GECI activity) in both strains. Graphs show the mean ± SEM from 6 assays (student’s t-test, ***p< 0,001.

Calcium in *T. gondii* is stored primarily in the acidocalcisomes and ER 11 and the latter organelle is a major site of PtdThr synthesis 2. We therefore presumed a role of PtdThr in regulating the calcium stores in the parasite ER. To confirm the notion, we performed additional assays using modulators of calcium signaling. Ethanol is an agonist of PLC, which regulates the IP3-sensitive calcium stores in the mammalian cells 23 as well as in *T. gondii* 10,24. We induced a maximum release of calcium to the parasite cytosol to estimate the total ion pool in the PTS mutant (Fig. 5A). Both, the parental and mutant strains expressing GECI showed equivalent fluorescence in the beginning of assays (0 min), which confirmed a comparable activity of functional GCaMP6s and vacuole numbers in both strains. We then observed a burst of EGFP signal in intracellular tachyzoites within 1 min of chemical exposure that remained constant thereafter. Even at maximal induction, fluorescence in the mutant’s cytosol was significantly less than the parental strain. Surprisingly, we also observed a much lower EGFP activity even after treatment of the Δ*tgpts* strain with ionophore A23187 (Fig. 5B), which should have equilibrated calcium across all membranes given its high concentration in culture medium (~ 1.8 mM). There was a slow but steady increase in signal following the initial surge that was probably due to continued flow of external calcium to the parasite interior. The results indicate that lower cytosolic fluorescence in the PTS mutant is likely caused by impaired storage of calcium in the parasite.

### Induced flux of calcium can repair egress and motility defects in the PTS mutant.

**Figure 6:**
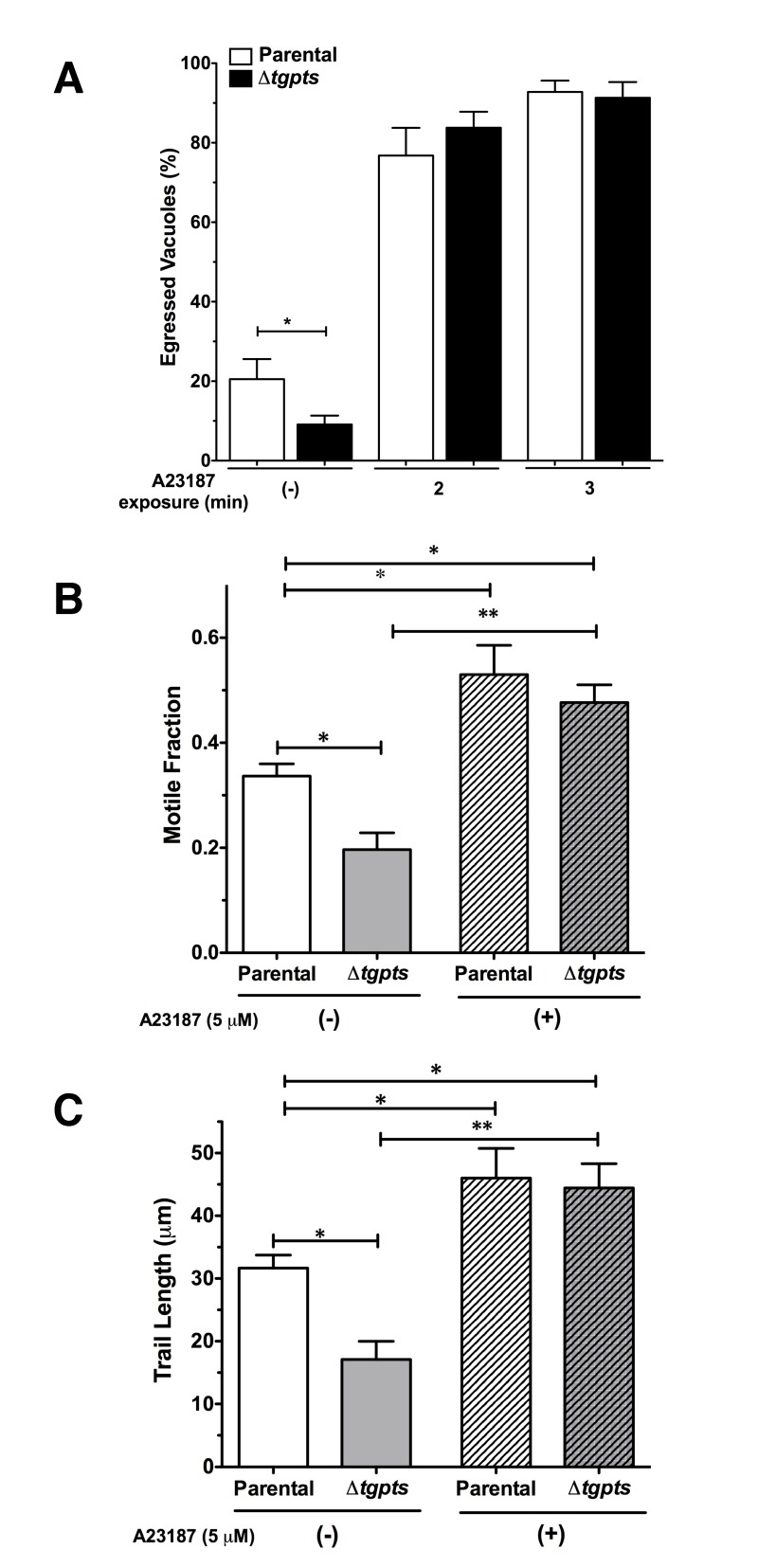
FIGURE 6: Exposure to a calcium ionophore can reinstate the egress and motility of the PTS-knockout strain. **(A)** Ionophore-induced egress in the parental or Δ*tgpts* strains. Parasitized cultures (MOI, 1; 48 h infection) were stimulated with 5 µM A23187 prior to fixation and staining. Percentage of egressed vacuoles was determined in at least 10 random fields at each time point. Note that the mutant exhibits a natural egress defect, which is amended within 2-3 min of drug exposure. **(B-C)** Motile fractions and trail lengths of the parasite strains treated with or without ionophore (5 µM A23187). About 500 parasites of each strain were analyzed for *Tg*Sag1 trails. Graphs in panel A-C show the mean ± SEM from 3 assays (student’s t-test, *p<0.05, **p<0.01).

We have previously shown that the Δ*tgpts* mutant lacking PtdThr shows a reversible defect in egress and gliding motility 2. To confirm whether these defects are indeed a consequence of perturbed calcium homeostasis in the mutant, we monitored the effect of ionophore on the parasite egress and motility. As shown in Fig. 6A, when intracellular Δ*tgpts* mutant was not stimulated with the ionophore, the strain showed a reduced egress compared to the parental strain. Whereas about 20% vacuoles were naturally disrupted in the parental strain, only 8% vacuoles exhibited parasite egress in the mutant 48 hours post-infection. Both strains revealed more than 90% egress and the egress defect in the mutant was no longer evident within 3 min of ionophore stimulation. Our motility assays showed that the motile fraction and trail lengths were markedly reduced in the mutant under control condition and exposure to ionophore significantly increased both parameters to a comparable level in the two strains (Fig. 6B-C). Likewise, caffeine, a pharmacological agonist of IP3/ryanodine-sensitive Ca^2+^ channels in *T. gondii* 24,25 also repaired gliding motility of the mutant (not shown). 

It is noteworthy that EGFP activity in ionophore-induced parental and mutant strains was much higher than the natural pools in the untreated samples (Fig. 4A and Fig. 5B). Just 1 min of exposure to ionophore triggered a prominent spike in fluorescence (Fig. 5B), which was greater than the apex ion level achieved in both strains under normal conditions (Fig. 4A). Such an elevated flux of calcium was indeed sufficient to restore egress and gliding motility in the mutant (Fig. 6). These results suggest a need of minimum calcium threshold for an efficient natural egress and gliding motility. Calcium signaling is also known to control exocytosis of micronemes, which actually underlies the motility and invasion 3,4,8,24,26,27. We did not observe a secretory defect in extracellular tachyzoites of the PTS mutant (not shown), which implies a lower threshold of calcium for microneme secretion. In this context, it should also be mentioned that microplate/cuvette-based quantification of the GECI signal in the extracellular stage have been difficult, presumably due to high variations in tachyzoite preparations and influence of milieu, which prevented us from studying reproducible oscillations in calcium during the secretion and invasion events. Future studies using more sensitive and color-tuned variants of GECI may elucidate the interrelationship of calcium, microneme exocytosis, gliding motility, egress and invasion processes. Equally, organelle-specific expression of GECIs shall illuminate PtdThr-mediated regulation of distinct calcium stores and their relative impacts on calcium signaling.

One of the several known functions of PtdSer, the natural and near universal analog of PtdThr, is to regulate calcium signaling and exocytosis in mammalian cells 28,29. In particular, PtdSer is known to facilitate the binding of membrane fusion machinery 30, alter the energy for the membrane bending 31, sense calcium to aid membrane fusion events during exocytosis 32, and regulate PLC- mediated IP3-dependent Ca^2+^ channels 33. In accord, PtdSer can also inhibit the slippage of calcium to the cytosol by sarcolemmal Ca^2+^-ATPase, and thereby increases the ion capture in the ER 34. Last but not least, synthetic derivatives of PtdThr containing defined acyl chains are more potent in inducing exocytosis than PtdSer species 35. It is therefore plausible that naturally occurring PtdThr can substitute for one or more of such functions of PtdSer in *T. gondii*. Along the line, the most predominant species of PtdThr (20:1, 20:5) present in tachyzoites 2 may serve as a source of arachidonic acid that can affect membrane fluidity and calcium flux 36. Although the mechanistic details remain to be examined, this work provides a stimulating premise on potential regulation of calcium homeostasis by PtdThr. Likewise, our method to quantify and compare cytosolic calcium within intracellular parasites as well as across the parasite strains should be applicable to other entwined models.

## MATERIALS AND METHODS

### Biological reagents and resources 

The RHΔ*ku80-hxgprt*- strain of *T. gondii* 37 was provided by Vern Carruthers (University of Michigan, USA). The Δ*tgpts* strain was generated in our earlier work 2. Anti-*Tg*Hsp90 19, anti-*Tg*Gap45 18 and anti-*Tg*Sag1 38 antibodies were donated by Sergio Angel (IIB-INTECH, San Martin, Argentina), Dominique Soldati-Favre (University of Geneva, Switzerland) and Jean-Francois Dubremetz (University of Montpellier, France), respectively. GCaMP6s 14 was obtained from Loren Looger (Howard Hughes Medical Institute, Ashburn, USA). Anti-GFP antibody and oligonucleotides were acquired from Life Technologies (Germany).

### Parasite and host cell cultures

Tachyzoites of all strains reported here were propagated in human foreskin fibroblast (HFF) cells in a humidified incubator (37°C, 5% CO2), as described previously 39. In brief, cells were cultured in Dulbecco's Modified Eagle Medium (DMEM) containing 10% fetal bovine serum, 2 mM glutamine, 100 µM of each MEM non-essential amino acids (glycine, alanine, asparagine, aspartic acid, glutamic acid, proline, serine), 1 mM sodium pyruvate, 100 U/ml penicillin and 100 µg/ml streptomycin. Parasites were cultured at a multiplicity of infection (MOI) of 3 every 2-3 days. HFF cells were harvested by trypsinization and grown to confluence in fresh flasks, dishes or plates as per experimental requirements.

### Molecular cloning and genetic manipulation of *T. gondii*

GCaMP6s was cloned into p*Tg*GRA1-UPKO plasmid at *Nsi*I/*Pac*I sites for expression under the control of the *Tg*GRA1 promoter and 3’UTR. GECI expression cassette was flanked by 5’ and 3’UTR of the *Tg*UPRT gene, which enabled targeted insertion at the UPRT locus. The complete sequence of GCaMP6s protein was amplified using CTCATCATGCATTCTCATCATCATCATCA TCATGG as the forward primer (*Nsi*I site) and CTCATCTTAATTAATTACTTCGCTGTCATCATTTGTACA as the reverse primer (*Pac*I site). Extracellular tachyzoites of the parental (RHΔ*ku80-hxgprt*-) and mutant (Δ*tgpts*) strains (~ 10^7^) were suspended in Cytomix (120 mM KCl, 0.15 mM CaCl_2_, 10 mM K_2_HPO_4_/KH_2_PO_4_, 25 mM HEPES, 2 mM EGTA, 5 mM MgCl_2_, pH 7.6), and then transfected using a BTX electroporation instrument (50 µg DNA, 2 kV, 50Ω, 25 µF, 250 µs). Parasites with a disrupted UPRT locus were selected by adding 5 µM of 5-fluorodeoxyuridine (FUDR) 40 and cloned by limiting dilution. It was not feasible to make an equivalent GCaMP6s-expressing PTS-complemented strain, which was also created by FUDR selection 2. Furthermore, the complemented strain exhibited unwarranted growth defect due to overexpression of PTS (not observed in the Δ*tgpts* mutant), which excluded its use for the assays reported in this work.

### Parasite phenotyping

All experiments were executed using fresh syringe-released extracellular tachyzoites. For plaque assays, 100-200 parasites of each strain were used to infect confluent HFF monolayers cultured in 6-well plates. Parasitized cells were incubated for 7 days without perturbation and then fixed with cold methanol followed by staining of plaques with crystal violet dye. Plaques were imaged and scored for their sizes and numbers using the ImageJ program (NIH, USA). For egress assays, HFF monolayers cultured on glass coverslips were infected with tachyzoites (MOI, 1; 40-72 h), as described elsewhere 41. Samples were subsequently fixed with 4% paraformaldehyde and 0.05% glutaraldehyde solution in PBS (2 min), neutralized by 0.1 M glycine/PBS (5 min) and blocked in 3% BSA/PBS (30 min). Egressed parasites were stained with anti-*Tg*Sag1 antibody (1:1500, 1 h). Cells were washed 3x with PBS, permeabilized with 0.2% Triton X-100/PBS (20 min), and stained with anti-*Tg*Gap45 antibody (1:3000, 1 h) to visualize parasites residing in the intact vacuoles. Samples were washed and stained with Alexa488/Alexa594-conjugated secondary antibodies (1:3000, 1 h). The fraction of egressed vacuoles was scored from the number of vacuoles with *Tg*Sag1-positive parasites. To examine the gliding motility, parasites were incubated on BSA (0.01%)-coated coverslips in Hank’s Balanced Salt Solution (15 min, 37°C). Samples were fixed with 4% paraformaldehyde and 0.05% glutaraldehyde for 10 min and stained with anti-*Tg*Sag1 and Alexa488 antibodies. The motile fractions and trail lengths were quantified using the ImageJ software.

### Immunofluorescence and immunoblot assays

GCaMP6s was localized either by EGFP imaging or by immunofluorescence assays. The method was essentially similar to egress assays except for that samples were permeabilized prior to incubation with antibodies. Anti-*Tg*Hsp90 (1:1000, rabbit) or anti-*Tg*Gap45 (1:6000, rabbit) antibodies were used together with anti-GFP antibody (1:5000, mouse) to examine the subcellular localization of GCaMP6s. Images were acquired using ApoTome microscope (Zeiss, Germany). To do immunoblot analysis, proteins were resolved by sodium dodecyl sulfate polyacrylamide gel electrophoresis (12%) and transferred to a nitrocellulose membrane (85 mA for 90 min) (AppliChem). The membrane was treated overnight with 5% skimmed dry milk (suspended in Tris-buffered saline and 0.2% and tween 20), incubated with anti-GFP (1: 1500, mouse) and anti-*Tg*Hsp90 (1:1000, rabbit) antibodies (1 h at room temperature), washed 3x for 5 min each, and then incubated with IR dyes (680 RD, 800 CW, each at 1: 10000 for 1 h). Proteins were visualized using Li-Cor imaging system. Densitometry analysis was done using Image studio software. Expression of GCaMP6s was normalized to the loading of *Tg*Hsp90 in each sample lane.

### Calcium measurements in intracellular parasites

HFF monolayers cultured in 24-well plates were infected with GCaMP6s-expressing parental and Δ*tgpts* strains (24-50 hrs) with an MOI of 3 and 3.75 to counterbalance for the invasion defect in the mutant 2. EGFP fluorescence in response to calcium was quantified using a microplate reader (BioTek Instruments; excitation, 485 nm; emission, 528 nm; area scan mode; 37°C). The uninfected (negative control) and infected cells exposed to 5 µM A23187 ionophore (positive control) were included alongside. Alternatively, we also performed live imaging using wide-field microscopy (Zeiss Axiovert 200M). EGFP signal was recorded for 1 min before adding ionophore (5 µM A23187) and then for 10 min after drug treatment. All assays were conducted in standard colorless culture medium containing ~1.8 mM calcium (without phenol red). No exogenous calcium or chelators were added during experiments. The pKa for calcium-bound and calcium-free GCaMP6s is ~6.2 and >9, respectively, and the pH titration curve is similar to that of EGFP 14. Our own experiments did not reveal an apparent sensitivity of GCaMP6s to the pH variation (pH 5-8).

## References

[1] Black MW, Boothroyd JC (2000). Lytic cycle of Toxoplasma gondii.. Microbiol Mol Biol Rev.

[2] Arroyo-Olarte RD, Brouwers JF, Kuchipudi A, Helms JB, Biswas A, Dunay IR, Lucius R, Gupta N (2015). Phosphatidylthreonine and Lipid-Mediated Control of Parasite
Virulence.. PLoS Biol.

[3] Carruthers VB, Sibley LD (1999). Mobilization of intracellular calcium stimulates microneme
discharge in Toxoplasma gondii.. Mol Microbiol.

[4] Vieira MC, Moreno SN (2000). Mobilization of intracellular calcium upon attachment of
Toxoplasma gondii tachyzoites to human fibroblasts is required for
invasion.. Mol Biochem Parasitol..

[5] Meissner M, Schlüter D, Soldati D (2002). Role of Toxoplasma gondii myosin A in powering parasite gliding
and host cell invasion.. Science.

[6] Lovett JL, Sibley LD (2003). Intracellular calcium stores in Toxoplasma gondii govern
invasion of host cells.. J Cell Sci.

[7] Garrison E, Treeck M, Ehret E, Butz H, Garbuz T, Oswald BP, Settles M, Boothroyd J, Arrizabalaga G (2012). A forward genetic screen reveals that calcium-dependent protein
kinase 3 regulates egress in Toxoplasma.. PLoS Pathog.

[8] Pace DA, McKnight CA, Liu J, Jimenez V, Moreno SNJ (2014). Calcium entry in Toxoplasma gondii and its enhancing effect of
invasion-linked traits.. J Biol Chem.

[9] Moudy R, Manning TJ, Beckers CJ (2001). The loss of cytoplasmic potassium upon host cell breakdown
triggers egress of Toxoplasma gondii.. J Biol Chem.

[10] Arrizabalaga G, Boothroyd JC (2004). Role of calcium during Toxoplasma gondii invasion and
egress.. Int J Parasitol.

[11] Moreno SNJ, Ayong L, Pace DA (2011). Calcium storage and function in apicomplexan
parasites.. Essays Biochem.

[12] Rohrbach P, Friedrich O, Hentschel J, Plattner H, Fink RHA, Lanzer M (2005). Quantitative calcium measurements in subcellular compartments of
Plasmodium falciparum-infected erythrocytes.. J Biol Chem.

[13] Borges-Pereira L, Budu A, McKnight CA, Moore CA, Vella SA, Hortua Triana MA, Liu J, Garcia CR, Pace DA, Moreno SN (2015). Calcium Signaling Throughout the Toxoplasma gondii Lytic
Cycle. A Study Using Genetically Encoded Calcium Indicators.. J Biol Chem.

[14] Chen T-W, Wardill TJ, Sun Y, Pulver SR, Renninger SL, Baohan A, Schreiter ER, Kerr RA, Orger MB, Jayaraman V, Looger LL, Svoboda K, Kim DS (2013). Ultrasensitive fluorescent proteins for imaging neuronal
activity.. Nature.

[15] Akerboom J, Chen T-W, Wardill TJ, Tian L, Marvin JS, Mutlu S (2012). Optimization of a GCaMP Calcium Indicator for Neural Activity
Imaging.. J Neurosci.

[16] Akerboom J, Carreras Calderón N, Tian L, Wabnig S, Prigge M, Tolö J, Gordus A, Orger MB, Severi KE, Macklin JJ, Patel R, Pulver SR, Wardill TJ, Fischer E, Schüler C, Chen TW, Sarkisyan KS, Marvin JS, Bargmann CI, Kim DS, Kügler S, Lagnado L, Hegemann P, Gottschalk A, Schreiter ER, Looger LL (2013). Genetically encoded calcium indicators for multi-color neural
activity imaging and combination with optogenetics.. Front Mol Neurosci.

[17] Nagai T, Sawano A, Park ES, Miyawaki A (2001). Circularly permuted green fluorescent proteins engineered to
sense Ca2+.. Acad Sci U S A.

[18] Plattner F, Yarovinsky F, Romero S, Didry D, Carlier MF, Sher A, Soldati-Favre D (2008). Toxoplasma Profilin Is Essential for Host Cell Invasion and
TLR11-Dependent Induction of an Interleukin-12 Response.. Cell Host Microbe.

[19] Echeverria PC, Figueras MJ, Vogler M, Kriehuber T, de Miguel N, Deng B, Dalmasso MC, Matthews DE, Matrajt M, Haslbeck M, Buchner J, Angel SO (2010). The Hsp90 co-chaperone p23 of Toxoplasma gondii: Identification,
functional analysis and dynamic interactome determination.. Mol Biochem Parasitol.

[20] Endo T, Sethi KK, Piekarski G (1982). Toxoplasma gondii: calcium ionophore A23187-mediated exit of
trophozoites from infected murine macrophages.. Exp Parasitol.

[21] Carruthers VB, Giddings OK, Sibley LD (1999). Secretion of micronemal proteins is associated with Toxoplasma
invasion of host cells.. Cell Microbiol.

[22] Mondragon R, Frixione E (1996). Ca(2+)-dependence of conoid extrusion in Toxoplasma gondii
tachyzoites.. J Eukaryot Microbiol.

[23] Hoek JB, Thomas AP, Rubin R, Rubin E (1987). Ethanol-induced mobilization of calcium by activation of
phosphoinositide-specific phospholipase C in intact
hepatocytes.. J Biol Chem.

[24] Lovett JL, Marchesini N, Moreno SNJ, Sibley LD (2002). Toxoplasma gondii microneme secretion involves intracellular
Ca(2+) release from inositol 1,4,5-triphosphate (IP(3))/ryanodine-sensitive
stores.. J Biol Chem.

[25] Chini EN, Nagamune K, Wetzel DM, Sibley LD (2005). Evidence that the cADPR signalling pathway controls
calcium-mediated microneme secretion in Toxoplasma gondii .. Biochem J.

[26] Lourido S, Shuman J, Zhang C, Shokat KM, Hui R, Sibley LD (2010). Calcium-dependent protein kinase 1 is an essential regulator of
exocytosis in Toxoplasma.. Nature.

[27] McCoy JM, Whitehead L, van Dooren GG, Tonkin CJ (2012). TgCDPK3 regulates calcium-dependent egress of Toxoplasma gondii
from host cells.. PLoS Pathog.

[28] Martin TW, Lagunoff D (1979). Inhibition of mast cell histamine secretion by N-substituteed
derivatives of phosphatidylserine.. Science.

[29] Martin TW, Lagunoff D (1979). Interactions of lysophospholipids and mast cells.. Nature.

[30] Zhang Z, Hui E, Chapman ER, Jackson MB (2009). Phosphatidylserine regulation of Ca2+-triggered exocytosis and
fusion pores in PC12 cells.. Mol Biol Cell.

[31] Zhang Z, Jackson MB (2010). Membrane bending energy and fusion pore kinetics in Ca2+
-triggered exocytosis.. Biophys J.

[32] Rogasevskaia TP, Churchward MA, Coorssen JR (2012). Anionic lipids in Ca2+-triggered fusion.. Cell Calcium.

[33] Lomasney JW, Cheng H-F, Kobayashi M, King K (2012). Structural basis for calcium and phosphatidylserine regulation of
phospholipase C δ1.. Biochemistry.

[34] Dalton Ka, Pilot JD, Mall S, East JM, Lee AG (1999). Anionic phospholipids decrease the rate of slippage on the
Ca(2+)-ATPase of sarcoplasmic reticulum.. Biochem J.

[35] Iwashita M, Makide K, Nonomura T, Misumi Y, Otani Y, Ishida M, Taguchi R, Tsujimoto M, Aoki J, Arai H, Ohwada T (2009). Synthesis and evaluation of lysophosphatidylserine analogues as
inducers of mast cell degranulation. Potent activities of
lysophosphatidylthreonine and its 2-deoxy derivative.. J Med Chem.

[36] Bonhomme A, Bouchot A, Pezzella N, Gomez J, Le Moal H, Pinon JM (1999). Signaling during the invasion of host cells by Toxoplasma
gondii.. FEMS Microbiol Rev.

[37] Huynh M-H, Carruthers VB (2009). Tagging of endogenous genes in a Toxoplasma gondii strain lacking
Ku80.. Eukaryot Cell.

[38] Dubremetz JF, Rodriguez C, Ferreira E (1985). Toxoplasma gondii: redistribution of monoclonal antibodies on
tachyzoites during host cell invasion.. Exp Parasitol.

[39] Gupta N, Zahn MM, Coppens I, Joiner KA, Voelker DR (2005). Selective disruption of phosphatidylcholine metabolism of the
intracellular parasite Toxoplasma gondii arrests its growth.. J Biol Chem.

[40] Donald RG, Roos DS (1995). Insertional mutagenesis and marker rescue in a protozoan
parasite: cloning of the uracil phosphoribosyltransferase locus from
Toxoplasma gondii.. Proc Natl Acad Sci U S A.

[41] Heaslip AT, Nishi M, Stein B, Hu K (2011). The motility of a human parasite, Toxoplasma gondii, is regulated
by a novel lysine methyltransferase.. PLoS Pathog.

